# Editorial: Venom peptides: A rich combinatorial library for drug development, volume II

**DOI:** 10.3389/fmolb.2023.1186828

**Published:** 2023-03-27

**Authors:** Denis Servent, Fernanda C. Cardoso, Maria Elena De Lima

**Affiliations:** ^1^ Université Paris-Saclay, CEA, Département Médicaments et Technologies pour La Santé (DMTS), SIMoS, Gif-sur-Yvette, France; ^2^ Institute for Molecular Bioscience, The University of Queensland, Brisbane, QLD, Australia; ^3^ Programa de Pós-Graduação em Medicina e Biomedicina, Santa Casa de Belo Horizonte, Belo Horizonte, Brazil; ^4^ Departamento de Bioquímica e Imunologia, Universidade Federal de Minas Gerais, Belo Horizonte, Brazil

**Keywords:** venoms, toxins, drug development, pharmacological tools, venomic, peptides

Peptide toxins are the main components of venoms and have been selected during the evolution process of venomous animals to specifically interfere with the vital physiological systems of their victims, a crucial step for prey capture or defence against predators (von Reumont et al., 2022). These small-reticulated and highly resistant peptides interact with high affinity and selectivity with their molecular targets and have been exploited as valuable pharmacological tools to study these receptors and ion channels or for therapeutic purposes (Cardoso, 2020; da Silva et al., 2022; Gilles and Servent, 2014; Herzig et al., 2020). About ten venom-derived drugs already reach the market to treat type-2 diabetes, chronic pain, hypertension or acute coronary syndrome and several dozen are in clinical trials and preclinical investigations (Fischer and Riedl, 2022). In the volume II of this Research Topic of Frontiers in Molecular Biosciences, we complete the state of the art of venoms research already published in volume I, by including seven new manuscripts describing venomics studies, pharmacological characterizations of toxins or preclinical development of venom-derived peptides isolated from arthropods, insects, acarians or amphibians.

It is well known that spider venoms represent a unique source of bioactive molecules with various potential medical applications in the treatment pain, stroke or ischemic injuries, and which are also exploited for their insecticidal properties. In their study, Cardoso et al., combine proteomic analysis and high-throughput ion channels assays to identify neuroactive toxins from *P. nigriventer* venom. This approach identifies potent modulators of Na_V_ and Ca_V_ channels as well as novel neuroactive peptides and paves the way for a holistic understanding of venom pharmacology.

Even if stings from wasps are often associated with severe clinical symptoms, their venoms have been less intensively studied than those of snakes, spiders, scorpions or cone snails. In order to decipher the venom composition and activity of the lesser banded wasp (*V. affinis*), Sunagar et al. performed proteotranscriptomic and biochemical analysis of this venom, highlighting the complexity of its enzymatic content (CAP, trypsin, hyaluronidase, phospholipase), associated with its highly defensive nature (hyperallergic effect).

Bombesin-like peptides (BLPs), derived from amphibian skin, have been shown to modulate multiple pharmacological effects on smooth muscles in various organs *via* their interactions with bombesin receptors (BB1, BB2, BB3). In their study, Zhang et al., identified two novel BLPs from the skin secretion of the hybrid frog, *Pelophylax kl. Esculentus* and performed their *ex vivo* pharmacological characterization. These peptides display no cytotoxic effect while they significantly increased contractions of the rat bladder and uterus *via* BB1 and/or BB2 receptors. These properties could be exploited in multiple diseases involving bombesin receptors.

Due to their interaction with bacterial cell membranes, amphiphatic and positively charged antimicrobial peptides (AMPs) may represent a therapeutic treatment for infections caused by antibiotic-resistant bacteria. To improve the *in vivo* potency of AMPs, Moreira Brito et al. showed that PEGylation of the LyeTx Ib spider-derived AMP increases its proteolytic resistance *in vitro* and reduced its toxicity against VERO cells, confirming the previous results *in vivo*. In addition, structural and protein interaction analyses of this peptide revealed i) the global conservation of its α-helical and amphipathic structure and ii) a slight variation in its association constants with anionic membranes as compared to non-PEGylated AMP. These results reinforce the great antimicrobial potential of this PEGylated AMP.

In their work investigating how rLosac and rLopap proteins derived from *Lonomia obliqua* bristles may affect the process of skeletal muscle regeneration, Alvarez et al., demonstrate that these two proteins stimulate myoblasts proliferation by modulating the activity of myogenic regulatory factors and the release of PGE_2_. In addition, these proteins possess anti-inflammatory property enhancing their protective potency in the regeneration of skeletal muscle after injury.

Bioactive peptides in venoms have been shown to affect various features of cancer such as cell proliferation, invasion, or migration, and some of these molecules are in preclinical development for cancer treatment. In their review, Lobba et al., describe i) the identification of Amblyomin-X from the transcriptome of the salivary gland of *Amblyomma sculptum* ticks, ii) its anticoagulant, antiangiogenic and antitumor effect and iii) how this molecule selectively induces apoptosis in cancer cells by inhibiting proteasome activity. Furthermore, in *in vivo* assays using different animal models, Amblyomin-X treatment promotes tumor regression and reduces metastasis, making it an attractive molecule for cancer therapy.

Interestingly, the same group published a manuscript in this Research Topic deciphering the structural and functional properties of the two domains of Amblyomin-X (Morais et al.). By combining solid-phase peptide synthesis, X-Ray crystallography and *in vitro* biological assays, the authors highlight the respective functional role of the two domains of the protein. The N-terminal domain, structured as a Kunitz-type peptide, supports the cytotoxicity of the Amblyomin-X in tumor cells, an effect that requires its internalization in targeted cells *via* its unstructured C-terminal region.

In conclusion, the research results that have been shown in this Research Topic reinforce the great potential of the molecules derived from different venoms as pharmaceutical drugs.
